# Investigating Continuance Intention for Telehealth Visits in Children’s Hospitals: Survey-Based Study

**DOI:** 10.2196/60694

**Published:** 2025-04-25

**Authors:** Sara Vannelli, Filippo Visintin, Simone Gitto

**Affiliations:** 1 Department of Industrial Engineering University of Florence Florence Italy; 2 Department of Information Engineering and Mathematics University of Siena Siena Italy

**Keywords:** telehealth visit, continuance intention, partial least squares structural equation modeling, PLS-SEM, children’s hospital

## Abstract

**Background:**

Telehealth visits are remote health care consultations conducted using digital technologies, such as video calls, phone calls, or web-based platforms. This type of service offers numerous benefits for both health care users and health care providers. Users save time and money by avoiding traveling to health care facilities. At the same time, health care providers can expand access to care for users in remote areas and enhance the continuity of care. These advantages are even more evident in pediatric settings, where attending in-person services must align with the commitments of the patient (eg, school activities) and the caregiver. Although the potential benefits of telehealth visits for users and health care providers were already known before the COVID-19 pandemic, its widespread adoption only occurred during it. Having experienced its benefits, hospitals are now, in the postpandemic phase, determined to maintain and strengthen their remote service offerings. It has, therefore, become crucial for them to understand the factors influencing users’ intention to continue using telehealth visits (or “continuance intention”), even now after the access restrictions to health care facilities imposed during the COVID-19 pandemic have been lifted. However, the literature lacks comprehensive, valid, and reliable models explaining users’ continuance intention toward telehealth visit services.

**Objective:**

This study aims to investigate the variables impacting users’ continuance intention toward telehealth visits and identify suggestions for improvement.

**Methods:**

Two models of variables impacting users’ continuance intention toward telehealth visits were developed. The first model applied to all users undergoing telehealth visits, while the second one applied only to patients who received a telehealth visit using videoconferencing tools. The models were created based on the literature and a qualitative study comprising interviews with physicians with extensive experience in telehealth visits. The models were then tested using partial least squares structural equation modeling on 477 responses obtained by administering a survey to guardians of patients who had received at least 1 telehealth visit in a major European children’s hospital.

**Results:**

Both models showed that the variable *information quality* positively influenced the variables *continuance intention* and *perceived usefulness* and that *perceived usefulness* positively influenced *continuance intention*. The first model was robust to the *medical specialty* and the *channel* used to deliver the visit. The second model also showed that *systems quality* positively influenced *information quality*.

**Conclusions:**

This study has identified and tested 2 comprehensive, valid, and reliable models on the variables influencing users’ continuance intention toward telehealth visits. Moreover, the study’s results provide insights for hospitals to improve telehealth visit services.

## Introduction

### Context and Motivation of the Study

Telehealth visits are medical consultations where physicians interact remotely (with audio and video or audio only) with patients in real time [1]. They benefit multiple stakeholders by saving travel time and money for patients [2,3], improving access to health care providers [4], allowing easier resource management and reducing facility costs [5], reducing clinical risks [6], and ensuring scalability [7] and continuity of care [8,9] without reducing health outcomes [10,11]. Although the potential benefits of telehealth visits have long been known, and this service has already been introduced in national and international regulations (eg, it was introduced in Italy >10 years ago [12]), its diffusion has been slower than expected [13]. It was only during the COVID-19 pandemic (hereafter pandemic) that the need to maintain social distancing resulted in telehealth visits becoming the primary means of access to care for many nonacute patients [14]. The pandemic forced policy makers, health care providers, and patients to overcome the main barriers to telehealth visit adoption [15]. For example, limited familiarity with telehealth visits and the tools required to access them has often been identified as a barrier to their use [16,17]. However, the pandemic compelled many patients and physicians to become more familiar with IT devices [18], and the act of making a video call has now become a part of the digital skills of many users. As policy makers have witnessed the benefits of telehealth visits, they are now pushing toward its widespread adoption (eg, Italy has planned huge investments in strengthening telehealth visits [19]). The consolidation of telehealth visits is a priority for health care organizations, given the increased familiarity acquired by physicians and patients with telehealth visit platforms and IT devices in general and the substantial investments in organizational transformation, infrastructure upgrades (including the acquisition of new equipment), and staff training. Now that the health risks associated with the pandemic are no longer a concern, the future diffusion of telehealth visits will significantly hinge on whether patients intend to continue using the service [20,21]. Hospitals must now leverage the experience gained to manage and improve this service [22,23]. This requires understanding the factors influencing users’ *continuance intention* (CINT) toward telehealth visits [24], that is, the users’ intention to continue using telehealth visits in the future after initial adoption [25].

Building on a systematic review of the literature and in-depth interviews with physicians, this work presents the results of a study aimed at identifying the factors influencing users’ CINT toward telehealth visits in a pediatric setting. Specifically, we proposed 2 models and tested them using partial least squares structural equation modeling (PLS-SEM) [26].

The pediatric setting is particularly interesting for conducting this type of study. Delivering telehealth visits in children’s hospitals is both appealing and challenging [1,27]. It is appealing because in-person visits could be inconvenient for at least 2 stakeholders (ie, children missing school and caregivers needing time off work), specialized facilities for children are scant outside major cities [28], and a telehealth visit facilitates continuity of care that is of utmost importance for pediatric patients [29]. It is challenging because surveying patient perceptions in pediatric settings requires the involvement of the patient’s parents or guardians (hereafter informal caregivers). The latter will make judgments based on their perceptions. Because separating the decisions of patients and informal caregivers is impossible in this setting, the term “users” will refer to both in the remainder of this paper.

The study was conducted in a major European children’s hospital, which started providing telehealth visits during the pandemic and was still providing them at the end of this study (February 2023).

### Literature Review, Research Gaps, and Research Questions

We conducted a systematic literature review on the Scopus database following the PRISMA (Preferred Reporting Items for Systematic Reviews and Meta-Analyses) guidelines [30]. The search, conducted in April 2022 and updated in October 2024, focused on journal articles, reviews, and conference proceedings published in English from 2016 to 2025. We used the query string (“telemedicine” OR “telehealth” OR “teleconsultation*” OR “televisit*” OR “mhealth” OR “m-health” OR “ehealth” OR “e-health”) AND (“pls-sem” OR “sem” OR “Structural equation modelling” OR “Structural equation modeling” OR “causal model*” OR “causal inference” OR “path analysis”) to search the “Title,” “Keywords,” and “Abstract” fields. In total, 626 articles were retrieved. After screening titles and abstracts for relevance, 73 articles were retained, with 6 removed for lack of access. These studies were reviewed, and those not focusing on users’ CINT toward telehealth visits or intention to use telehealth visits were discarded, resulting in 46 selected contributions. Two additional studies [31,32] were added through reference tracking. It is important to note that, from the analysis of the selected papers and the examination of the questionnaire items used in these studies, it emerged that authors sometimes use the terms “intention to use” or “behavioral intention” to refer to what is, in fact, a “CINT” (eg, [33-37]). This is particularly apparent in studies where it is specified that the questionnaires were administered to users with prior experience with telehealth services.

Table 1 summarizes the results of the review, highlighting in the “Unit of analysis” column whether the study focuses on telehealth visit services or a broader range of telehealth services (designated as “Multiple” in the table), including telehealth visits. It also specifies in the “Users” column the target groups of the services (eg, children, adults, and older adults), and in the “Experienced users” column, it specifies whether the study’s participants had prior experience with the service before taking part in the survey. Finally, the table lists the latent variables included in the models and the key theories (columns “Variables” and “Theories,” respectively) that were used or adapted in their development. Variables used to represent CINT, regardless of the name used, are shown in quotes in the “Variables” column. Looking at the studies (explicitly or implicitly) focusing on CINT, it can be noticed that several authors propose structural models, using constructs from the technology acceptance model (TAM), such as perceived usefulness (PU) [34,38-41] and perceived ease of use (PEU) [34,39,42]. PU is defined as the degree to which a user believes that using a technology would enhance their job performance or improve their efficiency in completing specific tasks, while PEU refers to the degree to which a user believes that using a technology would be free of effort. The TAM suggests that PEU and PU positively influence the intention to use, and PEU also positively influences PU. Existing literature on telehealth services has proven that PU [34,38,39] and PEU influence [39] CINT as well.

**Table 1:** Summary of studies presenting structural models related to users’ continuance intention toward telehealth visits or intention to use telehealth visits.

Reference	Unit of analysis	Users	Experienced users	Variables^a^	Theories
[43]	Multiple	Adults from ethnic minority group regions	Yes	Service quality, information quality, system quality, perceived usefulness, confirmation, satisfaction, and "continuance intention"	ECM^a^
[44]	Telehealth visit	Adults	Not controlled	Likelihood of seeking a telehealth visit if it is available, perceived ease of use, and perceived usefulness	TAM^b^
[45]	Telehealth visit	Millennials and Gen Z	Yes	Commitment to transformation, readiness to transformation, planned behavior, behavioral intention, and user experience	TPB^c^ and TRA^d^
[39]	Multiple	Adults	Yes	Self-motivation, social motivation, perceived ease of use, perceived usefulness, "behavioral intention", and use behaviors	TAM and SDT^e^
[46]	Telehealth visit	Adults	Not controlled	Intention to use, performance expectancy, effort expectancy, social influence, price value, and facilitating conditions	UTAUT2^f^
[47]	Multiple	Older adults	Not controlled	External variables, perceived ease of use, perceived risk, perceived usefulness, behavioral intention, and satisfaction	TAM
[42]	Telehealth visit	Adults	Yes	Wait time, visit length, patient-centered communication, perceived ease of use, secure, satisfaction, overall quality, "continuity", and future use	SCT^g^ and EMCMI^h^
[38]	Multiple	Adults	Yes	Health consciousness, health motivation, perceived technology accuracy, perceived critical mass, perceived privacy protection, perceived usefulness, perceived convenience, "intention to use", perceived value, and adoption	UTAUT^i^
[48]	Multiple	Patients with chronic diseases or in a subhealth status	Yes	Direct network penalty, cross-network externality, indirect network externality, confirmation, perceived value, satisfaction, "continuous adoption intention", habits, switching cost, and continuous adoption behavior	ECM
[33]	Multiple	Adults	Yes	Performance expectancy, effort expectancy, information quality, functionality, contamination avoidance, engagement, satisfaction, "behavioral intention", and personal innovativeness	UTAUT and D&M^j^
[34]	Multiple	Adults	Yes	eHealth literacy, perceived ease of use, perceived usefulness, attitude, social influence, and "intention to use"	TAM
[49]	Multiple	Adults	Yes	Propensity to trust, doctor characteristics (doctor’s ability and benevolence), risks (perceived privacy risk and physical risk), cognitive trust, emotional trust, "continuance intention", and positive word of mouth	SOR^k^
[50]	Multiple	Educated adults (graduate, postgraduate, and professionally qualified)	Yes	Functional barrier (use barrier, value barrier, and risk barrier), psychological barrier (tradition barrier and image barrier), purchase intention, brand love, "continuance intention", and trust	IRT^l^
[51]	Multiple	Older adults	Not controlled	Model based on UTAUT2 (performance expectancy, effort expectancy, social influence, facilitating conditions, hedonic motivation, price value, habit, service quality, trust, and government policy) and model based on the TAM (service quality, social influence, government policy, trust, hedonic motivation, price value, habit, and use intention)	UTAUT2 and TAM
[52]	Telehealth visit	Adults	Yes	Immediacy, telepresence, intimacy, substitutability, satisfaction, "continuance intention", and pandemic-induced anxiety	ISCM^m^ and PDT^n^
[40]	Multiple	Adults	Yes	eHealth literacy, perceived usefulness, information quality, system quality, service quality, expectation confirmation, customer satisfaction, subjective norm, and "continuance use intention"	ECM-ISC^o^
[35]	Multiple	Millennials and Gen Z	Yes	Performance expectancy, effort expectancy, social influence, price value, satisfaction, and "intention to use"	UTAUT2
[53]	Telehealth visit	Adults	Not controlled	Results demonstrability, compatibility, performance expectancy, effort expectancy, facilitating conditions, social influence, COVID-19, attitude, intention to use, perceived risk, perceived susceptibility to disease, perceived severity of disease, concerns for collection, concerns for secondary use, concerns for improper access, and concerns for errors	DOI^p^, UTAUT, HBM^q^, and CFIP^r^
[54]	Multiple	Adults with type 2 diabetes	Not controlled	Perceived usefulness, perceived ease of use, perceived safety, attitude toward using, and intention to use	TAM and PRT^s^
[55]	Telehealth visit	Adults	Yes	Attitude, performance expectancy, subjective norm, external facilitating conditions, internal facilitating conditions, autonomy, competence, relatedness, and motivation to use	SDT
[56]	Telehealth visit	Adults	Yes	Performance expectancy, effort expectancy, facilitating conditions, price value, contamination avoidance, functionality, engagement, satisfaction, information quality, and continuous use intention	SOR and UTAUT2
[57]	Telehealth visit	Adults	Yes	Social influence, perceived usefulness, perceived technology use risk, perceived ubiquity, health anxiety, offline consultation habit, perceived value, trust, and behavioral intention	SOR
[58]	Multiple	Adults	Not controlled	Use behavior, intention to use, perceived user adoption, geographic location, data privacy issue, resistance to use, personal experience, social influence, effort expectancy, performance expectancy, and hedonic motivation	UTAUT2
[36]	Multiple	Older adults	Yes	Use behavior, "behavioral intention", social influence, effort expectancy, performance expectancy, hedonic motivation, facilitating condition, price value, habit, quality of life, and service quality	UTAUT2
[59]	Multiple	Adults	Not controlled	Information quality, system quality, service quality, performance expectancy, effort expectancy, social influence, facilitating condition, hedonic motivation, price value, behavioral intention, habit, perceived severity, and use behavior	UTAUT2, D&M, and PMT^t^
[60]	Multiple	Older adults with chronic diseases	Yes	Effort expectancy, social influence, facilitating conditions, performance expectancy, confirmation, satisfaction, and "continuance intention"	ECM-ISC and UTAUT
[61]	Multiple	Adults	Yes	Personal values, social values, reasons for (relative advantage, trialability, compatibility, or observability), reasons against (complexity, aversion to change, or technological anxiety), attitude, intention to use, and domain-specific innovativeness	BRT^u^
[62]	Multiple	Adults	Not controlled	Health consciousness, health motivation, perceived compatibility, perceived critical mass, perceived usefulness, perceived technology accuracy, perceived privacy protection, intention to use, perceived value, and adoption	UTAUT
[63]	Multiple	Adults	Yes	Internal health locus of control, performance expectancy, effort expectancy, social influence, and intention to use	UTAUT and HLOC^v^
[37]	Multiple	Adults in self-quarantine during the pandemic	Yes	Performance expectancy, effort expectancy, social influence, facilitating condition, hedonic motivation, habit, price value, health consciousness, "behavioral intention", actual use behavior, self-quarantine, and mental well-being	UTAUT2
[64]	Multiple	Adults	Yes	Contamination avoidance, safety, reliability, professionalism, perceived ease of use, perceived usefulness, information quality, facilitating conditions, social influence, behavioral intention to use, and actual use	TAM
[32]	Telehealth visit	Adults	Not controlled	Self-efficacy, personal innovativeness, availability, contamination avoidance, effort expectancy, social influence, habit, performance expectancy, facilitating conditions, intention to use, and perceived risk	UTAUT2
[65]	Telehealth visit	Adults	Not controlled	Perceived risk, personal innovativeness, perceived ease of use, perceived usefulness, and behavioral intention	TAM, PRT, and PIT^w^
[66]	Telehealth visit	Adults taking general medical treatment from hospitals	Not controlled	Performance expectancy, effort expectancy, social influence, facilitating conditions, perceived vulnerability, perceived severity, response efficacy, information quality, system quality, services quality, computer self-efficacy, intention to adopt, and attitude	UTAUT, D&M, and PMT
[67]	Multiple	Adults	Not controlled	Social influence, technology anxiety, trust, perceived risk, perceived physical condition, resistance to change, perceived usefulness, perceived ease of use, and attitude	TAM
[68]	Multiple	Children	Not controlled	Performance expectancy, effort expectancy, social influence, facilitating condition, hedonic motivation, price value, and behavioral intention	UTAUT2
[69]	Multiple	Adults without mental disorders capable of communicating	Not controlled	Performance expectancy, effort expectancy, social influence, perceived risk, facilitating conditions, and behavioral intention	UTAUT
[41]	Multiple	Adults	Yes	Perceived usefulness, confirmation, satisfaction, "continuance intention", motivation, competence, autonomy, and relatedness	ECM-ISC and SDT
[37]	Multiple	Adults	Yes	Performance expectancy, effort expectancy, social influence, facilitating conditions, perceived reliability, price value, gender, "behavioral intention", and actual use behavior	UTAUT
[31]	Multiple	Adults with chronic diseases	Not controlled	Use intention, privacy, trust, perceived usefulness, perceived ease of use, social influence, facilitating conditions, technological anxiety, users’ resistance to technology, and perceived risk	TAM
[70]	Multiple	Patients with chronic diseases	Not controlled	Perceived outcome, perceived information risk, emotional preference, perceived medical liability, perceived convenience, attitude, health consciousness, behavioral intention, perceived medical risk, subjective norm, perceived behavioral control, and perceived severity of disease	TPB
[71]	Telehealth visit	Adults	Yes	Quality, expectation confirmation, trust, usefulness, and "continuance intention"	None
[72]	Multiple	Adults	Not controlled	Trust, perceived risks, perceived usefulness, perceived ease of use, and intention to adopt	TAM
[73]	Multiple	Adults	Not controlled	Perceived usefulness, perceived ease of use, privacy, trust, intention to use, gender, and actual use	TAM
[74]	Multiple	Older adults	Not controlled	Performance expectancy, effort expectancy, social influence, facilitating condition, behavioral intention, technology anxiety, resistance to change, and use behavior	UTAUT
[75]	Multiple	Older adults	Not controlled	Performance expectancy, effort expectancy, facilitating conditions, social influence, doctor’s opinion, computer anxiety, perceived security, and behavioral intention to use	UTAUT
[76]	Multiple	Young citizen	Not controlled	Perceived usefulness, perceived ease of use, subjective norm, innovativeness, intention to use, gender, and actual use	TAM2^x^
[25]	Multiple	Adults	Yes	Platform quality, quality of advice, interaction quality, perceived value, satisfaction, "continuance intention", and quality of health life	WANG^y^

^a^ECM: expectation confirmation model.

^b^TAM: technology acceptance model.

^c^TPB: theory of planned behavior.

^d^TRA: theory of reasoned action.

^e^SDT: self-determination theory.

^f^UTAUT2: extended unified theory of acceptance and use of technology.

^g^SCT: social cognitive theory.

^h^EMCMI: ecological model of communication in medical interactions.

^i^UTAUT: unified theory of acceptance and use of technology.

^j^D&M: DeLone and McLean information success model.

^k^SOR: stimulus-organism-response framework.

^l^IRT: innovation resistance theory.

^m^ISCM: information systems continuance model.

^n^PDT: psychological distance theory.

^o^ECM-ISC: extended expectation confirmation model of information systems continuance.

^p^DOI: diffusion of innovation theory.

^q^HBM: health belief model.

^r^CFIP: concerns for information privacy framework.

^s^PRT: perceived risk theory.

^t^PMT: protection motivation theory.

^u^BRT: behavioral reasoning theory.

^v^HLOC: internal health locus of control.

^w^PIT: personal innovativeness theory.

^x^TAM2: extended technology acceptance model.

^y^WANG: Wang information systems success model.

Quality-related constructs, too, are frequently used in studies investigating CINT toward telehealth visits. Quality is either considered as a global aspect of performance [42,71] or split into more fine-grained components [25,33,36,40,43,56]. In this second case, authors usually resort to the characterization proposed in the DeLone and McLean information systems success model (D&M model) [77]. The D&M model considers *information quality* (INF_Q), *systems quality* (SYS_Q), and *service quality* (SERV_Q). INF_Q measures the system’s success in conveying the intended meaning, SYS_Q measures the desired characteristics of the system used to provide the service, and SERV_Q measures the overall support delivered by the service provider.

While studies examining a broader set of telehealth services or multipurpose platforms could provide valuable insights, their findings cannot be generalized to telehealth visits, as the services they consider could significantly differ from telehealth visits (eg, Megawati et al [45] also consider services such as buying medicines, booking laboratory services, and reading articles). Surprisingly, only 4 studies investigating CINT toward telehealth services specifically focus on telehealth visits [42,52,56,71]. However, 3 of these studies [42,52,56] present models designed for specific contexts, limiting their generalizability to other settings. For example, Amin et al [56] and Lu et al [52] focused on CINT in resource-limited countries and included variables such as “price value” and “pandemic-induced anxiety.” These variables are irrelevant in contexts such as Italy, where telehealth visit services are priced as in-person ones at least in public hospitals, and the pandemic is no longer a concern. Wu and Brannon [42] instead focused on chronic patients and their focus was on “patient-centered communication.” This construct, however, is relevant in the context of patients receiving severe diagnoses (eg, cancer), who must endure considerable emotional distress; navigate complex medical information; and make challenging, life-altering treatment decisions, which is not the case in the context under study (we considered pediatric patients with minor health issues; refer to the Methods section). Finally, Grenier Ouimet et al [71] considered the *quality* of telehealth visits as a formative variable composed of *ease of use*, *service quality*, and *security and confidentiality* and demonstrated that *quality* influences CINT. However, conceptualizing *quality* as a formative variable means that the indicators cause the constructs, implying that omitting an indicator potentially alters the nature of the construct and reduces the generalizability of results [78]. Furthermore, it contradicts the literature, which suggests studying quality-related aspects separately [77,79]. In addition, they collected data on patients who had telehealth visits before the pandemic, arguing that their sample is more indicative of CINT in a postpandemic setting because survey responses collected during the crisis are unlikely to be representative of behavior under normal conditions. However, we argue that examining users who were introduced to telehealth visit during the pandemic represents a crucial complementary dimension. These patients, compelled to experience a new model of care, may have discovered the benefits of telehealth visits that they would not have otherwise experienced and may show a higher propensity to reuse it.

Finally, only 1 study [68] has investigated telehealth services in pediatric settings. This study, however, does not focus on CINT, considers a broad set of telehealth services, and applies the Extended Unified Theory of Acceptance and Use of Technology (UTAUT2) model without introducing any new variables or relationships and dismissing the significance of half of the tested causal relationships.

In summary, the literature lacks comprehensive, valid, and reliable models explaining the CINT toward telehealth visit services that can be applied in pediatric settings. Nevertheless, it provides an abundance of theories, constructs, and relationships that can guide the creation of relevant models. This study, thus, aims to answer the following research question: What factors influence CINT toward a telehealth visit in pediatric settings?

## Methods

### Overview

To answer the aforementioned research question, this study involved a qualitative and a quantitative phase. The qualitative phase involved conducting 8 in-depth interviews with physicians who were purposefully sampled based on their extensive experience with telehealth visits. The quantitative phase involved developing and evaluating 2 PLS-SEM models on variables impacting users’ CINT toward telehealth visits.

### Qualitative Phase: Interviews With Physicians

Eight in-depth interviews with physicians with extensive experience with telehealth visits were conducted.

The interviews had 3 key objectives: understanding the criteria physicians apply to assess patient eligibility for telehealth visit (hereafter referred to as “eligibility criteria”), obtaining insights into physicians’ perspectives on the factors influencing CINT, and fostering physicians’ engagement in the study by actively involving them in the subsequent survey administration process.

We created a concise interview guide featuring open-ended questions designed to explore physicians’ experiences with telehealth visits. Verbal informed consent was obtained from all participants at the start of each interview. Each session lasted approximately 30 minutes and occurred between June and July 2022. Physicians were asked about their medical specialty, the duration of their practice in the field, and the eligibility criteria they used. They were also invited to describe the typical process of a telehealth visit, beginning with proposing the option to a patient and concluding with the session’s end, any challenges they have faced during these visits, the feedback they have received from patients, and whether patients have expressed concerns or complaints about the service. Finally, they were asked to give their opinion on the factors that could incentivize users to continue using telehealth visits after having tried it.

### Quantitative Phase: Building the Models, Data Collection, and Testing the Models

#### Building the Models

Two models were developed based on the literature and the interviews. Interviews were transcribed and coded. Using open-ended questions allowed us to gather information from the respondents in an unbiased and nonleading way [80]. The interviews were transcribed verbatim and abductively coded, starting from the variables identified in the literature review. Two researchers performed the coding and classification process independently. They first created a list of factors whose definitions were derived from the literature, and they then used them as a framework for coding physicians’ responses. Whenever the 2 coders disagreed about classification factors, they discussed their reasons with a third researcher until consensus was established.

**Figure 1:**
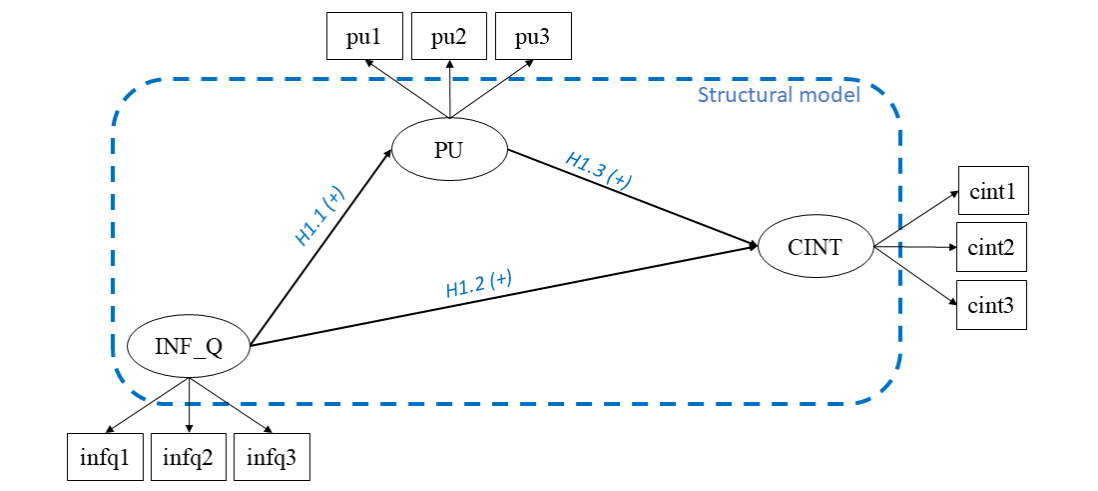
Model 1, which was applied to all users undergoing telehealth visits. CINT: continuance intention; H1.1: hypothesis 1.1; H1.2: hypothesis 1.2; H1.3: hypothesis 1.3; INF_Q: information quality; PU: perceived usefulness.

**Figure 2:**
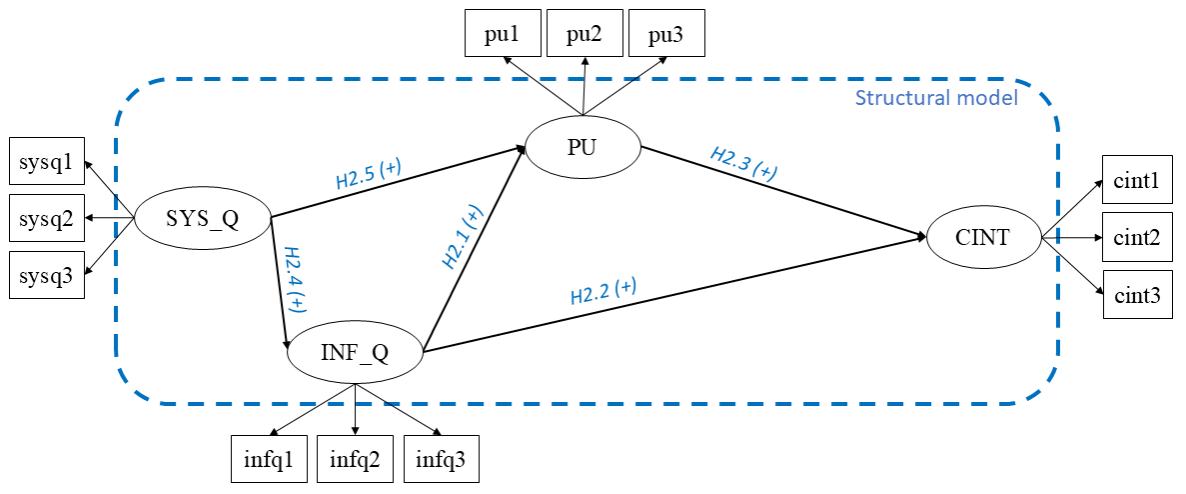
Model 2, which was applied only to those receiving telehealth visits using videoconferencing tools (ie, users visited by telephone were ruled out). CINT: continuance intention; H2.1: hypothesis 2.1; H2.2: hypothesis 2.2; H2.3: hypothesis 2.3; H2.4: hypothesis 2.4; H2.5: hypothesis 2.5; INF_Q: information quality; PU: perceived usefulness; SYS_Q: system quality.

The first model (Figure 1) applies to all users undergoing telehealth visits, and the second one (Figure 2) applies only to those receiving telehealth visits using videoconferencing tools (ie, users visited by telephone were ruled out). The hypotheses proposed by this study are grounded on both success models for information systems (ISs) [77,79] and the TAM [81-83].

The 2 models are thoroughly described in the Results section.

Given the lack of models in the literature that were well suited to the context under study, specialists’ opinions proved invaluable in identifying the variables to be included in the models. For example, because items reflecting the quality of the conveyed information (INF_Q) emerged from the interviews as influencing CINT, we decided to include the variable in the models.

Moreover, because the eligibility criteria used in the study hospital—including selecting patients without severe or complex-to-diagnose medical conditions, residing in areas with adequate telephone coverage, not requiring a language interpreter, and having an established relationship with the physician—were shown to align with both the literature [4,84-87] and national regulations [12,19,88], taking these criteria into account enabled us to exclude variables from the models that, while frequently used in the literature, would have been homogeneous within our sample. For example, *technology anxiety*, *personal innovativeness*, or *eHealth literacy* were omitted because physicians do not schedule telehealth visits for users who lack the technical skills required to interact remotely, and *trust* was excluded because physicians do not schedule telehealth visits for users with whom they do not have an established relationship. This approach allowed for the development of more parsimonious and contextually relevant models.

The interviews also informed the measurement model (ie, questionnaire indicators that reflect the latent variables in the models). Indeed, we first drafted the measurement scales looking at the literature (Table 2), but the scales were subsequently adapted to incorporate the physicians’ comments. For example, thanks to the interviews, we understood which system characteristics were relevant to measure the system quality (SYS_Q) in the specific context. Then, in line with state-of-the-art guidelines [89-91], we validated the questionnaire’s validity, completeness, and readability with 2 physicians, the hospital medical director, and 1 staff member. They were presented with the latent variables and the measurement models and asked whether the questionnaire items were clear and captured the most relevant aspects to measure the variables. On the basis of the collected feedback, we slightly revised the questions’ wording without significantly changing, adding, or removing any indicators. For privacy reasons, we could not interact directly with users throughout the research, so the validation of the questionnaire was conducted with people with considerable experience related to telehealth visit processes and who had received feedback from users over time. The questionnaire was designed to be short. The header of the questionnaire reassured respondents of the anonymity and confidentiality of the study (no personal data were collected). The wording of the questions was designed to avoid creating a sense that certain responses were correct or incorrect. Reducing ambiguities and inconsistencies, minimizing respondents’ efforts, protecting respondent anonymity, and reducing evaluation apprehension reduced the *common method bias* [92].

**Table 2:** Operationalization of the research variables.

Construct_ID and item_ID		Item	Scale	Relevant PLS-SEM^a^ or SEM^b^ studies	
**AGE_CAR^c^**					—^d^
	age_car	Age of the parent or guardian of the patient who received the telehealth visit	Ordinal scale		
**AGE_PAT^e^**					—
	age_pat	Age of the patient	Ordinal scale		
**SPEC^f^**					—
	spec	Indicate for which medical specialty you made your last telehealth visit: (1) dermatology, (2) diabetology, (3) endocrinology, (4) gastroenterology, (4) immunology, or (5) other	Nominal scale		
**CHAN^g^**					—
	chan	The telehealth visit was conducted via (1) videoconference or (2) telephone call	Nominal scale		
**SYS_Q^h^**					[40,59]
	sysq1	I saw the physician clearly	Five-point interval Likert-type scale		
	sysq2	I heard the physician clearly	Five-point interval Likert-type scale		
	sysq3	The telehealth visit platform (log-in, file sharing, and audio and video features) is simple	Five-point interval Likert-type scale		
**INF_Q^i^**					[40,56,59,64]
	infq1	I believe that the telehealth visit, in my case, was as effective as an in-person visit	Five-point interval Likert-type scale		
	infq2	I effectively conveyed my health needs to the physician during the telehealth visit	Five-point interval Likert-type scale		
	infq3	I believe that the duration of the telehealth visit was adequate	Five-point interval Likert-type scale		
**PU^j^**					[44,71,93]
	pu1	The telehealth visit allowed me to save time (eg, travel and waiting time)	Five-point interval Likert-type scale		
	pu2	The telehealth visit allowed me to save money (eg, train ticket, gasoline, or parking costs)	Five-point interval Likert-type scale		
	pu3	The telehealth visit allowed me to better combine the visit with my home and work commitments	Five-point interval Likert-type scale		
**CINT^k^**					[40,41,71,94]
	cint1	I would recommend the telehealth visit to friends and relatives	Five-point interval Likert-type scale		
	cint2	I would like to undergo a telehealth visit again	Five-point interval Likert-type scale		
	cint3	I am overall satisfied with the telehealth visit	Five-point interval Likert-type scale		

^a^PLS-SEM: partial least squares structural equation modeling.

^b^SEM: structural equation modeling.

^c^AGE_CAR: age of the informal caregiver.

^d^Not applicable.

^e^AGE_PAT: patient’s age.

^f^SPEC: medical specialty.

^g^CHAN: delivery channel.

^h^SYS_Q: systems quality.

^i^INF_Q: information quality.

^j^PU: perceived usefulness.

^k^CINT: continuance intention.

#### Data Collection

After consolidating the final wording, the 8 physicians who participated in the interviews sent the link to the survey by email to all users who had had at least 1 telehealth visit with them from January 2021 to June 2022. The population who received the email comprised 2650 users. The survey was administered using Google Forms and ensured the anonymity of the respondents. The administration period lasted 4 months (November 2022-February 2023), during which we obtained 477 responses (477/2650, 18% response rate). The administration of the questionnaire was entrusted to the physicians for two reasons: (1) due to privacy concerns, it was not possible to access any patient data; and (2) it was believed that a request coming from a physician familiar with the patients would be more likely to receive a response.

It is worth pointing out that in Italy, telehealth visits can only replace follow-up visits [12]; therefore, all users undergo at least 1 in-person visit before receiving a telehealth visit. This approach enabled us to survey users who (1) had experienced both modes of delivery, allowing for direct comparison, and (2) were deemed eligible for telehealth visits by physicians who had previously treated them.

#### Testing the Models

Data collected were checked to detect *unengaged responses* and *outliers*, while *missing* data were not a concern as all answers were mandatory. Data cleaning led us not to discard any response. Finally, the models were tested using PLS-SEM [26]. The state-of-the-art guidelines in PLS-SEM analysis and result reporting [78,95,96] were followed.

### Ethical Considerations

This study did not involve clinical trials, the processing of sensitive patient data, medical record consultation, or sample archiving. According to national and regional regulations (Decreto Legislativo n. 211/2003, Legge Regione Toscana n. 40/2005, Legge Regione Toscana n. 84/2015), ethics committee approval is not required for this type of research. The hospital management formally authorized the study. The research was conducted in accordance with the Declaration of Helsinki and followed Good Clinical Practice guidelines. Verbal informed consent was obtained from all interviewees prior to participation. Participants were informed about the voluntary nature of the interview, the purpose of the study, and how their data would be used and stored. For the questionnaire, respondents were informed via email—sent by a physician with whom they had previously interacted during a telehealth visit—that participation was voluntary. Completion of the questionnaire was considered as implied consent to participate, in line with standard ethical practices for anonymous surveys. All interviews were anonymized, and no identifying information was collected or stored. The identity of interviewees is not disclosed at any point in the publication. Questionnaire responses were collected anonymously. The authors had no means to associate responses with individual users, and the design of the questionnaire (closed-ended questions only) further prevented the inclusion of personal information. Data collection, processing, and management complied fully with national and European data protection regulations (EU Regulation No. 679/2016 and Decreto Legislativo No. 101/2018). Participants (both interviewees and survey respondents) received no compensation or incentives for their participation. No identifiable features or personal data of participants are included in this publication. All data were anonymized at the point of collection and handled in accordance with relevant data protection regulations.

## Results

### Included Variables

This paragraph presents and discusses the variables included in the models considering the results of the interviews with physicians. The dependent latent variable in our models, CINT, assesses users’ willingness to continue using telehealth visits in the future and their likelihood of recommending the service to others.

The results of the qualitative study proved the importance of quality-related aspects in explaining CINT. Indeed, interviewees frequently cited items reflecting them as influencing CINT.

Moreover, the interviews confirmed the appropriateness of the D&M model to study quality-related aspects in the case of telehealth visits in a pediatric setting. The quality types proposed in the D&M model were contextualized by considering the telehealth visit service’s characteristics and the interviews’ results. A telehealth visit requires *direct* and *remote* interaction between physicians and users. This interaction happens either via phone or via a video call application. The system used to deliver the service does not convey any additional relevant information other than that exchanged between the user and the physician (users can, at most, upload a medical report or download a prescription). In this context, the D&M model’s quality types can be interpreted as follows:

- SYS_Q depends on how easily the system can be accessed and its ability to guarantee seamless audio and video communication and file sharing. In our study, SYS_Q refers
  to the degree to which the videoconferencing platform was easy to use and allowed for smooth audio-video interaction.
- INF_Q relates to the user’s perceptions of the quality of the information exchanged with the physician during telehealth visits. The accuracy of the conveyed information, that is, the accuracy of the received diagnosis or the effectiveness of the prescribed treatment, is a “credence” property [97], and as such, it can only be evaluated by users after months of treatment [98]. As a result, INF_Q is influenced more by the quality of the communication with the physician than by the specific content of the information provided. Thus, in our study, *INF_Q* captures *the extent to which the user, through direct and remote interaction, could convey what they would have expressed in person, receive feedback comparable to that of face-to-face interaction, and engage with the physician for an adequate amount of time*.
- SERV_Q holds minimal relevance, at least in the investigated setting, and was not included in the models. Interviews indicated that there were no instances of system downtime during the analysis period nor was there any need for technical support (users could take the system availability for granted).

Interviewees cited benefits regarding time, money, and professional-personal life balance as factors impacting CINT. They are always cited as the most relevant benefits for users [2,3] as using telehealth visits has proven not to negatively impact health outcomes [10,11]. These benefits reflect the PU variable in the TAM (corresponding to the performance expectancy in the Unified Theory of Acceptance and Use of Technology [UTAUT] and UTAUT2 [99,100]). In our models, PU measures *how a telehealth visit* *enables users to save time or money or better integrate the visit into their home and work commitments.*

### Proposed Hypotheses

Here, we present the 2 hypothesized models. In model 1, a total of 3 reflective latent variables were included: PU, INF_Q, and CINT. We introduce 3 hypotheses:

- Hypothesis 1.1, INF_Q positively influences PU.
- Hypothesis 1.2, INF_Q positively influences CINT.
- Hypothesis 1.3, PU positively influences CINT.

We considered 2 control variables: *patient’s age* (AGE_PAT) and the *age of the informal caregiver* (AGE_CAR). Moreover, we assessed the model robustness to 2 categorical variables, ie, the *delivery channel* (CHAN; ie, audio-video or audio only) through which the telehealth visit is provided and the *medical specialty* (SPEC) for which the telehealth visit was conducted (which depends on the patient’s health issues). The impact of CHAN [101-103] and SPEC [104,105] on stakeholders involved in telehealth visits has been widely cited in the literature, but their impact on CINT has not been studied quantitatively.

In *model 2*, a fourth latent variable, SYS_Q, was added.

The sample for model 2 was restricted to users who had been visited by a videoconferencing platform (164 in total). As the videoconference visits require using a web-connected device and a videoconferencing platform, we were interested in understanding whether the SYS_Q of this tool affected the other latent variables. Conversely, SYS_Q was ruled out in model 1 because if users had any difficulty interacting via telephone, they would not be considered eligible for a telehealth visit.

Model 2 has 5 hypotheses:

- Hypothesis 2.1, INF_Q positively influences PU.
- Hypothesis 2.2, INF_Q positively influences CINT.
- Hypothesis 2.3, PU positively influences CINT.
- Hypothesis 2.4, SYS_Q positively influences INF_Q.
- Hypothesis 2.5, SYS_Q positively influences PU.

As for model 1, we considered AGE_PAT and AGE_CAR as control variables.

Hypothesis 1.1 and hypothesis 2.1 (INF_Q positively influences PU) and hypothesis 2.5 (SYS_Q positively influences PU) have been proposed and tested by studies on telehealth services based on IS success models [25,59] and by others based on the TAM [31,34,39,47,54,65,67,73]. Indeed, the hypotheses are grounded on both IS success models [77,79] and the TAM [81-83]. The D&M model proposes an association between the 3 types of quality and *net benefits* (ie, the balance of positive and negative impacts of the system on users, suppliers, employees, organizations, markets, industries, etc). Considering the main benefits a telehealth visit offers users, PU is associated with the *net benefits* variable in the D&M model. Similarly, in the context of a telehealth visit, the D&M model’s SYS_Q and TAM’s PEU constructs largely overlap.

Hypothesis 1.2 and hypothesis 2.2 (INF_Q positively influences CINT) are coherent with the D&M model [77] and qualitative studies on telehealth visits [106,107]. Moreover, they have been proposed and tested by studies on telehealth services [33,56].

Hypothesis 1.3 and hypothesis 2.3 (PU positively influences CINT) are supported by existing literature on telehealth services [25,34,37,39-41,43,56,60,71] and are coherent with the literature on the IS success model [77,79].

Hypothesis 2.4 (SYS_Q positively influences INF_Q) has been proposed in the literature on telehealth visits [106,107]. However, to the best of the authors’ knowledge, this relationship has not yet been quantitatively tested. While prior studies have investigated the independent effects of quality types, they have largely overlooked the relationships between them. Beyond the telehealth context, studies exploring relationships between quality dimensions indicate that when users perceive a higher quality in the system delivering the service (SYS_Q), their perception of the information quality (INF_Q) also improves [108]. Hypothesis 2.4 is based on the idea that if the system’s quality is not high, the system does not guarantee seamless communication and information sharing with the physician.

### Measurement Scales

This paragraph presents the measurement scales used to test the hypothesized models.

To measure the latent reflective variables in models 1 and 2, we used multi-indicator measurement scales comprising 3 indicators for all the variables (SYS_Q, INF_Q, PU, and CINT). Our questionnaire contains 18 items for users who have received a telehealth visit via videoconferencing and 15 items for those who have received a telehealth visit via telephone. The indicator list and the corresponding variables are listed in Table 2. All the indicators are derived from the measurement scales used in PLS-SEM or structural equation modeling (SEM) studies (refer to the column “Relevant PLS-SEM or SEM studies” in Table 2). However, they have been modified based on feedback from interviewed physicians and insights from relevant studies on telehealth visits [41,109-113].

### Results of Testing the Models

#### Overview

The measurement models were verified by checking *internal consistency*, *convergent validity*, *discriminant validity*, *collinearity*, and *overfitting* [78,95,96,114]. *Internal consistency* was checked by resorting to the Cronbach α and the Composite Reliability (CR) index. *Convergent validity* was assessed by looking at the average variance extracted (AVE) and the estimated indicator loadings. Estimated indicator loadings indicate the strength of the relationship between reflective latent variables and their indicators. We checked their practical relevance and statistical significance. *Discriminant validity* was verified by looking at the heterotrait-monotrait ratio of correlations (HTMT) matrix, which is calculated as the correlations of indicators across constructs measuring different phenomena relative to the average of the correlations of indicators within the same construct [115].

For each model, we checked the *path hypotheses*, looking at the estimated path coefficients, the *collinearity* among latent variables, and the *overfit*. The path coefficients represent the strength and direction of the relationships between latent variables. We checked that the direction of the relationships was as hypothesized and the relationships were statistically significant. *Collinearity* among latent variables was assessed by looking at variance inflation factors (VIFs). *Overfit* was assessed by examining the coefficient of determination (*R*^2^).

Then, we checked the robustness of the structural models to categorical variables (ie, SPEC and CHAN) after checking the partial measurement invariance of variables through the Measurement Invariance of COMposite models (MICOM) procedure [116]. The procedures comprise three hierarchically interrelated steps: (1) *configural invariance*, (2) *compositional invariance*, and (3) *the equality of variables’* *mean value and varianc*e*.* *Configural invariance* is a qualitative assessment of whether variables are equally parametrized and estimated between groups. *Compositional invariance* tests whether the scores of the latent variables across groups are correlated. In the third step, we examine whether the mean values and variances of the latent variables in the first group and those obtained in the second group differ. Partial measurement invariance occurs when both configural and compositional invariance are verified. Full measurement invariance, instead, occurs when partial measurement invariance is verified and the variables have equal mean values and variances across the groups. At least partial measurement invariance must be verified to compare the path coefficient estimates of the structural relationships between the variables across the groups [116,117]. To check the robustness of the structural models to categorical variables, we applied a distribution-free approach based on an approximate random test [96,118].

#### Model 1

In model 1, *internal consistency* was guaranteed (α and CR were >0.7). The AVE was >0.5 (Table S1 in Multimedia Appendix 1), while the estimated indicator loadings were all practically relevant (|estimated indicator loading|≥0.70) and statistically significant (Table 3). The HTMT matrix (Table S2 in Multimedia Appendix 1) allows us to consider the CINT-PU and PU-INF_Q as conceptually different (HTMT<0.85), while CINT-INF_Q is slightly outside the acceptable threshold for discriminant validity (HTMT<0.90).

**Table 3:** Model 1—estimated indicator loadings.

Indicator loading	Estimated indicator loading
INF_Q^a^=~infq1	0.918^b^
INF_Q=~infq2	0.860^b^
INF_Q=~infq3	0.833^b^
PU^c^=~pu1	0.912^b^
PU=~pu2	0.810^b^
PU=~pu3	0.933^b^
CINT^d^=~cint1	0.942^b^
CINT=~cint2	0.879^b^
CINT=~cint3	0.953^b^

^a^INF_Q: information quality.

^b^*P*<.001.

^c^PU: perceived usefulness.

^d^CINT: continuance intention.

All the path hypotheses (hypothesis 1.1-hypothesis 1.3) are validated (Table 4). Both control variables are not significant (*P*=.56 for value for CINT~AGE_CAR and *P*=.98 for CINT~AGE_PAT). Collinearity is excluded (VIF<3; Table S3 in Multimedia Appendix 1). Moreover, the variables in the model explained 50% of the variance on PU and 87% on CINT, and the overfit was excluded as *R*^2^<0.9 (Table S4 in Multimedia Appendix 1).

We tested the robustness of model 1 to 2 qualitative variables: CHAN and SPEC.

CHAN discriminates against 2 groups: users who have been visited via teleconference (n=164) and those who have been visited by telephone (n=313). Configural and compositional invariance across groups is verified, and no significant differences emerge in the estimated path coefficients (Table 5), while the equality of the variables’ mean value and variance is not verified. Users who received a telehealth visit by telephone reported, on average, a lower level of CINT and a higher level of PU, while no significant differences emerged in terms of INF_Q across groups (Table 6).

SPEC discriminates between 3 groups: dermatology (n=215), gastroenterology (n=196), and others (n=66). In addition, in this case, only partial measurement invariance is verified, and no significant differences emerge in the estimated path coefficients (Table 5). Dermatology users reported, on average, a higher level of both CINT and PU than others, while no significant differences emerged in INF_Q (Table 6).

**Table 4:** Model 1—estimated path coefficients.

Path	Hypothesis	Estimated path coefficients
PU^a^~INF_Q^b^	1.1	0.708^c^
CINT^d^~INF_Q	1.2	0.674^c^
CINT~PU	1.3	0.323^c^

^a^PU: perceived usefulness.

^b^INF_Q: information quality.

^c^*P*<.001.

^d^CINT: continuance intention.

**Table 5:** Model 1—estimation of the structural model by groups.

Grouping variable, groups, and path				Estimated path coefficient							Difference				
				G1		G2		G3		G1 − G2			G1 − G3		G2 − G3
**CHAN^a^**															
	**G1: videoconference; G2: telephone**														
		PU^b^~INF_Q^c^	0.679^d^		0.723^d^		—^e^		−0.045			—		—	
		CINT^f^~INF_Q	0.702^d^		0.703^d^		—		−0.002			—		—	
		CINT~PU	0.285^d^		0.302^d^		—		−0.017			—		—	
**SPEC^g^**															
	**G1: dermatology; G2: gastroenterology; G3: others**														
		PU~INF_Q	0.718^d^		0.706^d^		0.707^d^		0.012			0.012		−0.001	
		CINT~INF_Q	0.714^d^		0.714^d^		0.675^d^		−0.0003			0.039		0.039	
		CINT~PU	0.274^d^		0.302^d^		0.331^d^		−0.029			−0.057		−0.029	

^a^CHAN: delivery channel.

^b^PU: perceived usefulness.

^c^INF_Q: information quality.

^d^*P*<.001.

^e^Not applicable.

^f^CINT: continuance intention.

^g^SPEC: medical specialty.

**Table 6:** Model 1—descriptive statistics of the variables in groups and differences between groups.

Grouping variable, groups, and variable				Mean (SD)							Difference of the variable’s mean value								
				G1		G2		G3		G1 − G2			G1 − G3		G2 − G3				
**CHAN^a^**																			
	**G1: videoconference; G2: telephone**																		
		INF_Q^b^	3.750 (1.125)		3.653 (1.180)		—^c^		0.0830			—		—					
		PU^d^	4.417 (1.016)		4.101 (1.220)		—		0.2697^e^			—		—					
		CINT^f^	3.892 (1.150)		3.390 (1.325)		—		0.390^g^			—		—					
**SPEC^h^**																			
	**G1: dermatology; G2: gastroenterology; G3: others**																		
		INF_Q	3.722 (1.126)		3.600 (1.228)		3.823 (1.064)		0.103			0.056		−0.047					
		PU	4.352 (1.041)		4.003 (1.304)		4.359 (1.005)		0.291^e^			0.216^i^		−0.075					
		CINT	3.763 (1.213)		3.310 (1.374)		3.662 (1.149)		0.344^e^			0.277^g^		−0.068					

^a^CHAN: delivery channel.

^b^INF_Q: information quality.

^c^Not applicable.

^d^PU: perceived usefulness.

^e^*P*=.008

^f^CINT: continuance intention.

^g^*P*=001.

^h^SPEC: medical specialty.

^i^*P*=.033.

#### Model 2

The measurement for model 2 was also verified; α>0.7 and CR>0.7 confirmed *internal consistency* (Table S5 in Multimedia Appendix 1), while an AVE≥0.5 (Table S5 in Multimedia Appendix 1) coupled with practically relevant (|estimated variable loading| ≥0.70) and statistically significant estimated indicator loadings (Table 7) ensured *convergent validity*. *Discriminant validity* is verified as HTMT<0.85 for all pairs of variables (Table S6 in Multimedia Appendix 1), except for CINT-INF_Q (HTMT<0.90).

Hypotheses 2.1 to 2.4 are validated, while hypothesis 2.5 is not (Table 8). A VIF<3 allows excluding collinearity (Table S7 in Multimedia Appendix 1). Moreover, the variables in the model explained 49% of the variance in PU, 43% of the variance in INF_Q, and 86% of the variance in CINT. Overfit is excluded as well (*R*^2^<0.9; Table S8 in Multimedia Appendix 1).

**Table 7:** Model 2—estimated indicator loadings.

Indicator loading	Estimated variable loading
SYS_Q^a^=~sysq1	0.796^b^
SYS_Q=~sysq2	0.781^b^
SYS_Q=~sysq3	0.705^b^
INF_Q^c^=~infq1	0.854^b^
INF_Q=~infq2	0.815^b^
INF_Q=~infq3	0.851^b^
PU^d^=~pu1	0.930^b^
PU=~pu2	0.766^b^
PU=~pu3	0.949^b^
CINT^e^=~cint1	0.937^b^
CINT=~cint2	0.869^b^
CINT=~cint3	0.967^b^

^a^SYS_Q: systems quality.

^b^*P*<.001.

^c^INF_Q: information quality.

^d^PU: perceived usefulness.

^e^CINT: continuance intention.

**Table 8:** Model 2—estimated path coefficients.

Path	Hypothesis	Estimated path coefficient
PU^a^~INF_Q^b^	2.1	0.526^c^
CINT^d^~INF_Q	2.2	0.699^c^
CINT~PU	2.3	0.287^c^
INF_Q~SYS_Q^e^	2.4	0.654^c^
PU~SYS_Q	2.5	0.232

^a^PU: perceived usefulness.

^b^INF_Q: information quality.

^c^*P*<.001.

^d^CINT: continuance intention.

^e^SYS_Q: systems quality.

## Discussion

### Insights From a Managerial Perspective

With regard to model 1, we verified all the path hypotheses. We verified hypothesis 1.1 (INF_Q positively influences PU), which indicates that when patients perceive the information conveyed through a telehealth visit as high quality, they are more likely to perceive the time savings, cost reduction, and enhanced convenience that the telehealth visit offers. Therefore, it is crucial for users to feel well attended and have their concerns thoroughly addressed; otherwise, the benefits of telehealth visits may not be fully appreciated.

We verified hypothesis 1.2 (INF_Q positively influences CINT): users can assess the clinical outcomes of a telehealth visit only after months of treatments [98], so their CINT depends on whether they feel that they can effectively communicate their needs, doubts, and concerns to the physician and receive attention for sufficient time and that an in-person visit would not have been more effective. It is, therefore, essential to focus not only on the accuracy of the diagnosis or prescription—something users may not always be able to evaluate—but also on the process through which it is delivered. For instance, physicians must not appear hasty even when the diagnosis is straightforward. Similarly, physicians should be careful when squeezing telehealth visits between various daily commitments, as rushing could impact users’ perception of the conveyed information.

We verified hypothesis 1.3 (PU positively influences CINT): as telehealth visits have proven as effective as in-person visits from a clinical standpoint [10,119], CINT is influenced by the time, costs, and inconvenience users can avoid by being visited remotely [15,112,120]. Thus, in evaluating the appropriateness of assigning a user to a telehealth visit, it is essential to consider not just the geographic distance from the study hospital but also variables such as the user’s work schedule, responsibilities for caring for other family members, and potential limitations in mobility as they could influence CINT.

No significant difference in model 1 is found between the 2 groups differing in CHAN, meaning that the delivery channel (teleconference or telephone) does not influence the magnitude of the identified causal relationships. On average, users who receive a telehealth visit by telephone were less inclined to continue using the telehealth visit. The lower CINT may be due to a greater empathy established through an audio-video channel than the telephone [121,122]. Considering this result, audio-video calls should be preferred to phone calls when possible (ie, when users can rely on a reliable device and connection). Surprisingly, on average, users who receive a telehealth visit by telephone perceived less time and cost savings (PU). This could be explained by the fact that when an appointment is made for a telephone visit, physicians are less careful to consider patients’ needs and take their availability for granted. This can result in some inconvenience for users. Therefore, if telehealth visits are assigned, the user’s availability should not be taken for granted to not create inconvenience for users. Finally, no significant differences in INF_Q were observed between patients visited by telephone and those visited by teleconference. This probably implies that before assigning users a telehealth visit by telephone, physicians had ensured that the channel was appropriate to convey the information the telehealth visit was supposed to convey.

No difference in the structural model emerged between the 3 groups created by SPEC. However, on average, users who receive a telehealth visit for dermatological issues are more willing to continue using the telehealth visit and perceive higher time and cost savings than the ones who have received a telehealth visit for another medical specialty. These results could be explained by the fact that most dermatologic pathologies are diagnosed with the naked eye and require frequent consultations [107]. As dermatology obtained a higher CINT, hospitals may consider incentivizing the use of telehealth visit for this specialty.

With regard to model 2, we verified the path hypotheses 2.1 (INF_Q positively influences PU), 2.2 (INF_Q positively influences CINT), and 2.3 (PU positively influences CINT), similarly as for model 1.

We verified hypothesis 2.4 (SYS_Q positively influences INF_Q). Hence, if the videoconferencing platform is user friendly and allows for smooth audio-video interaction, users will more likely perceive the quality of the visit as adequate. This suggests paying close attention to the platform’s user-friendliness. For example, hospitals that want to use their own platforms (eg, to make it easier to report on activities performed remotely) need to ensure that these solutions do not involve significant effort on the user’s part.

We did not verify hypothesis 2.5 (SYS_Q positively influences PU). This means that the platform’s user-friendliness does not influence the PU of telehealth visits.

### Conclusions

In this study, we adopted a mixed methods approach to propose and test 2 PLS-SEM models that explored the key variables influencing CINT toward a telehealth visit in children’s hospitals. The variables were contextualized through an initial qualitative phase and grounded in established theories, including IS success models [77,79] and the TAM [81,83,99]. We proposed 2 comprehensive yet parsimonious models, avoiding overcontextualized variables and ensuring the validity and reliability of our findings by following state-of-the-art guidelines for constructing and testing measurement and structural models. This study addresses a significant gap in existing literature, which features context-specific models and findings that may not be applicable to children’s hospitals in the postpandemic era. Although several studies have provided valuable insights into telehealth visit adoption, their findings are often confined to health care settings that differ from pediatric care. For instance, some studies focus on developing countries and investigate telehealth visit use during the pandemic [52,56], when emergency circumstances drastically altered health care delivery. These studies do not capture the more stable, long-term integration of telehealth visits into children’s hospitals in developed economies. The study by Wu and Brannon [42] instead focused on adult patients with chronic conditions and patient-centered communication. This study emphasizes the complexities of telehealth visit communication in contexts where patients may require a high level of emotional support in addition to medical advice. This is not the case in pediatric settings where telehealth visit services are only reserved for patients with minor ailments. Finally, some studies rely on prepandemic data [71], which fail to account for the rapid technological advancements, shifts in patient expectations, and changes in regulatory environments that have emerged since the pandemic. This study, therefore, fills in this gap by providing insights specific to the current postpandemic context and to children’s hospitals, where telehealth is increasingly being integrated into routine care rather than being viewed as a stopgap measure.

From a managerial perspective, health care service providers and policy makers could draw insights from this study to answer the wake-up call of taking proactive actions to improve telehealth visits [123]. The findings highlight the critical role of INF_Q in shaping users’ perceptions of the benefits of telehealth visits, such as time savings, cost reduction, and convenience. They highlight the importance of a thoughtful delivery process, urging physicians to avoid appearing rushed, even when providing straightforward information, and to consider users’ unique circumstances, such as work commitments, caregiving responsibilities, and mobility limitation—in addition to the geographic distance from the hospital—when deciding to offer telehealth visit services to patients. The study also suggests that audio-video calls should be preferred over phone calls when feasible and that close attention should be paid to the platform’s user-friendliness. Finally, they suggest that although it may be easier to reach patients by phone rather than scheduling a videoconference, this should not lead to assuming patients’ availability when scheduling phone visits.

Our results are generalizable to other children’s hospitals because the eligibility criteria that guided the choice of variables in our models are widely used and consistent with the literature [4,84-87] and national regulations [12,19,88].

This paper is not without limitations. In the survey, we asked users to recall their last telehealth visit. It could have created a *recall bias* [90] for some users due to the time gap between filling out the survey and their telehealth visit. Furthermore, the time elapsed since the last telehealth visit could vary from patient to patient. In addition, due to privacy concerns, we could not interview hospital patients directly. Instead, factors influencing CINT were inferred indirectly through interviews with physicians (who, however, had conducted thousands of telehealth visits and had informally gathered substantial feedback over time). Moreover, this study is based only on data from 1 hospital. Administering the survey in different hospitals with different telehealth visit platforms would have allowed for greater external validity of our findings and better investigation and inclusion of platform-related factors in the model.

In addition to healing these problems, future studies could incorporate variables taken from other models into the models explaining users’ CINT toward telehealth visits. For example, users’ *expectations*, borrowed from the Expectation Confirmation Model, could be included. This would allow for a deeper understanding of how the alignment or misalignment between initial expectations and the actual experience of using a telehealth visit (*confirmation*) influences satisfaction and, consequently, CINT.

In addition, in this study, we deliberately decided to exclude SERV_Q—one of the quality dimensions included in the D&M model—from our model. This decision was based on the assumption, supported by empirical evidence, that the technical infrastructure underlying modern telehealth visit platforms is consistently reliable. In our case, users could rely on the infrastructure’s availability and functionality; indeed, no instances of system downtime occurred during the period of analysis. However, in our context, the technologies (video call platforms and smartphones) used to deliver telehealth visits were mature, and the patients considered eligible for this service had reliable internet connections and sufficient familiarity with the technology. When these conditions are not met, the quality of the health care provider’s support could play a crucial role in influencing CINT, making it important to include SERV_Q in the model.

## Appendix

Multimedia Appendix 1 Tables S1 to S8. Supplementary results on models 1 and 2.

## Abbreviations

AGE_CAR: age of the informal caregiver
AGE_PAT: patient’s age
AVE: average variance extracted
CHAN: delivery channel
CINT: continuance intention
CR: Composite Reliability
D&M model: DeLone and McLean information systems success model
HTMT: heterotrait-monotrait ratio of correlations
INF_Q: information quality
IS: information system
PEU: perceived ease of use
PLS-SEM: partial least squares structural equation modeling
PRISMA: Preferred Reporting Items for Systematic Reviews and Meta-Analyses
PU: perceived usefulness
SPEC: medical specialty
SYS_Q: systems quality
TAM: technology acceptance model
UTAUT: unified theory of acceptance and use of technology
UTAUT2: extended unified theory of acceptance and use of technology
VIF: variance inflation factor
