# Supplementary material for: Investigating Continuance Intention for Telehealth Visits in Children’s Hospitals: Survey-Based Study
Source: J Med Internet Res. 2025 Apr 25;27:e60694. doi: 10.2196/60694 (PMC12064977; doi:10.2196/60694)
Supplement: Multimedia Appendix 1 [file jmir_v27i1e60694_app1.docx]

Table S1: Model 1 - AVE, Cronbach’s alpha, and CR

| **Variable** | **AVE** | **Cronbach’s alpha** | **CR** |
| --- | --- | --- | --- |
| PU | 0.786 | 0.916 | 0.917 |
| INF_Q | 0.759 | 0.904 | 0.904 |
| CINT | 0.856 | 0.947 | 0.947 |

Table S2: Model 1 - HTMT matrix

|  | **PU** | **INF_Q** | **CINT** |
| --- | --- | --- | --- |
| **PU** | 1 | 0 | 0 |
| **INF_Q** | 0.708 | 1 | 0 |
| **CINT** | 0.802 | 0.903 | 1 |

Table S3: Model 1 – VIF

| **Dependent variable** | **Independent variable** | **VIF** |
| --- | --- | --- |
| CINT | PU | 2.037 |
|  | INF_Q | 2.011 |

Table S4: Model 1- R^2^ of dependent variables

| **Dependent variable** | **R^2^** |
| --- | --- |
| PU | 0.502 |
| CINT | 0.871 |

Table S5: Model 2 - AVE, Cronbach’s alpha, and CR

| **Variable** | **AVE** | **Cronbach’s alpha** | **CR** |
| --- | --- | --- | --- |
| SYS_Q | 0.581 | 0.804 | 0.806 |
| PU | 0.785 | 0.915 | 0.916 |
| INF_Q | 0.706 | 0.878 | 0.878 |
| CINT | 0.856 | 0.946 | 0.947 |

Table S6: Model 2 - HTMT matrix

|  | **SYS_Q** | **PU** | **INF_Q** | **CINT** |
| --- | --- | --- | --- | --- |
| **SYS_Q** | 1 | 0 | 0 | 0 |
| **PU** | 0.578 | 1 | 0 | 0 |
| **INF_Q** | 0.654 | 0.676 | 1 | 0 |
| **CINT** | 0.680 | 0.766 | 0.893 | 1 |

Table 7: Model 2 – VIF

| **Dependent variable** | **Independent variable** | **VIF** |
| --- | --- | --- |
| PU | SYS_Q | 1.749 |
|  | INF_Q | 1.749 |
| CINT | PU | 1.917 |
|  | INF_Q | 1.890 |

Table 8: Model 2- R^2^ of dependent variables.

| **Dependent variable** | **R^2^** |
| --- | --- |
| INF_Q | 0.428 |
| PU | 0.491 |
| CINT | 0.852 |
